# Supplementary figures and images for: Sex-Specific Whole-Transcriptome Analysis in the Cerebral Cortex of FAE Offspring
Source: Cells. 2023 Jan 15;12(2):328. doi: 10.3390/cells12020328 (PMC9856965; doi:10.3390/cells12020328)

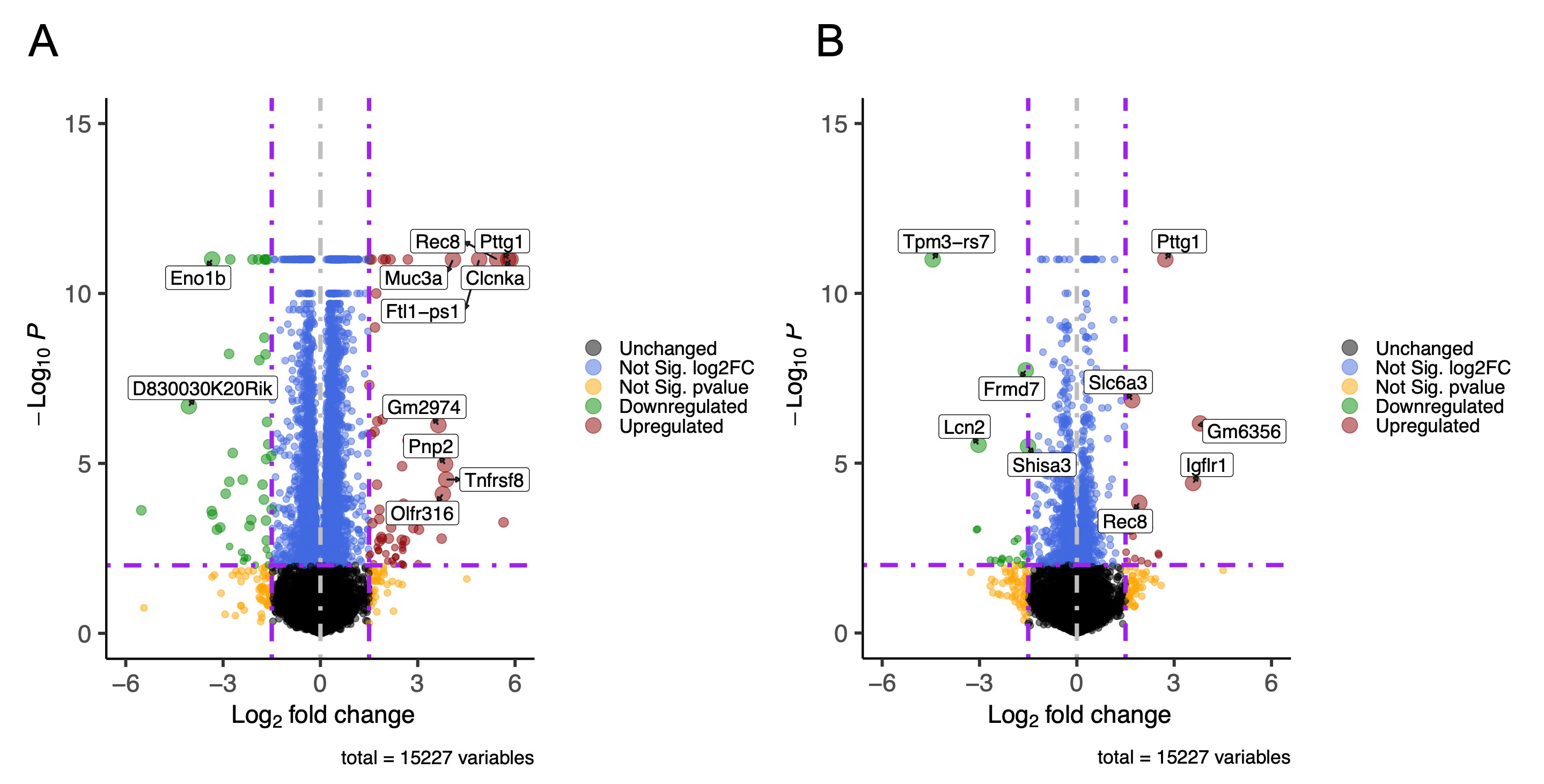

Supplement: Supplementary file 1 [file cells-12-00328-s001.zip › Figure S1.jpg]

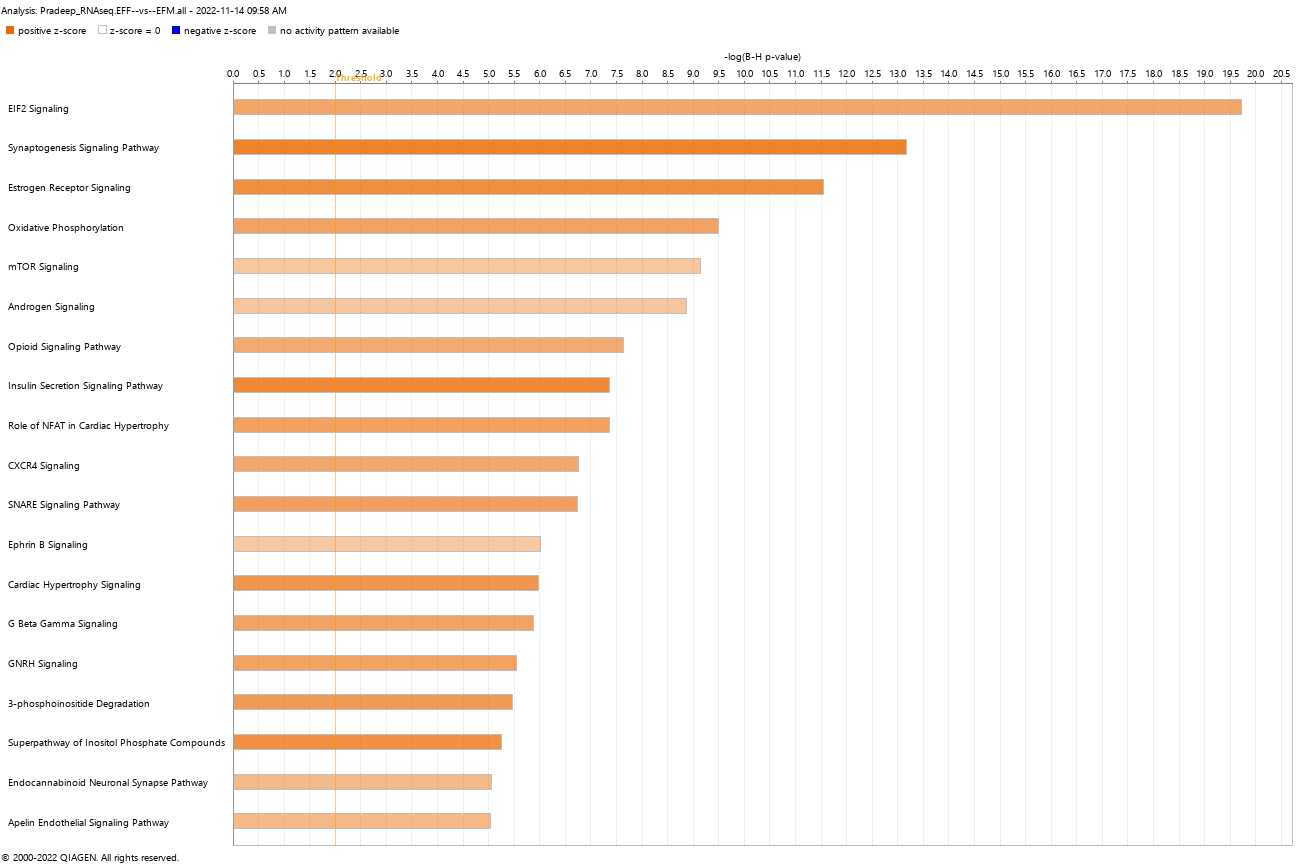

Supplement: Supplementary file 1 [file cells-12-00328-s001.zip › Figure S2.jpg]
